# Neuroprotective effects of resveratrol in Alzheimer disease pathology

**DOI:** 10.3389/fnagi.2014.00218

**Published:** 2014-09-11

**Authors:** Shraddha D. Rege, Thangiah Geetha, Gerald D. Griffin, Tom L. Broderick, Jeganathan Ramesh Babu

**Affiliations:** ^1^Department of Nutrition, Dietetics, and Hospitality Management, Auburn UniversityAuburn, AL, USA; ^2^Department of Chemistry, Auburn University at MontgomeryMontgomery, AL, USA; ^3^Department of Biology, Tuskegee Institute, Tuskegee UniversityAL, USA; ^4^Laboratory of Diabetes and Exercise Metabolism, Department of Physiology, Midwestern UniversityGlendale, AZ, USA

**Keywords:** Alzheimer disease, beta–amyloid, oxidative stress, resveratrol, neuroprotection

## Abstract

Alzheimer’s disease is a chronic neurodegenerative disorder characterized by a progressive loss of cognitive and behavioral abilities. Extracellular senile plaques and intracellular neurofibrillary tangles are hallmarks of AD. Researchers aim to analyze the molecular mechanisms underlying AD pathogenesis; however, the therapeutic options available to treat this disease are inadequate. In the past few years, several studies have reported interesting insights about the neuroprotective properties of the polyphenolic compound resveratrol (3, 5, 4′-trihydroxy-*trans*-stilbene) when used with *in vitro* and *in vivo* models of AD. The aim of this review is to focus on the neuroprotective and antioxidant effects of resveratrol on AD and its multiple potential mechanisms of action. In addition, because the naturally occurring forms of resveratrol have a very limited half-life in plasma, a description of potential analogs aimed at increasing the bioavailability in plasma is also discussed.

## INTRODUCTION

**FIGURE 1:**
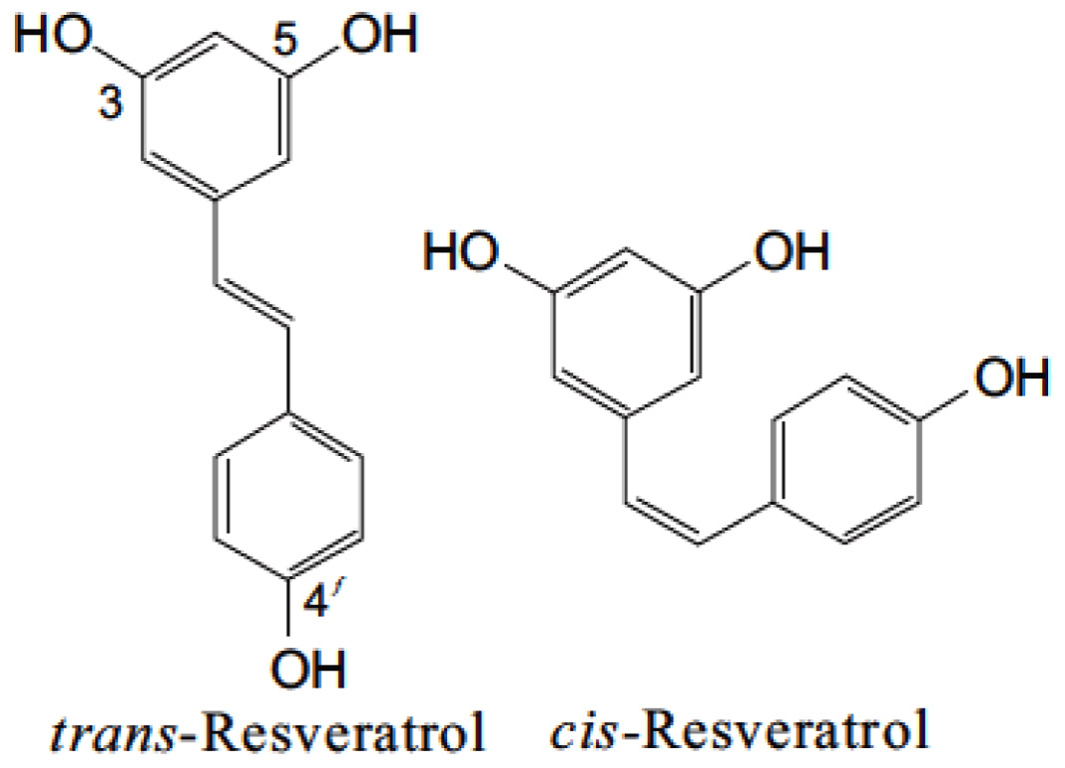
**Isomers of resveratrol.** Chemical structures of *trans* and *cis*-resveratrol.

Resveratrol (3,5,4′-trihydroxy-*trans*-stilbene) is a naturally occurring polyphenolic compound, which belongs to the phytoalexin superfamily. This compound was first isolated from the roots of white hellebore (*Veratrum grandiflorum O. LOES*) and was named by Dr. Michio Takaoka in his thesis in 1940. The discovery of resveratrol by Dr. Takaoka was the prime step leading to establishing the scientific efficacy of the Chinese “material medica,” a collection of traditional Asian medicines (Takaoka, 1940). In 1963, resveratrol was isolated from the roots of *Polygonum cuspidatum*, a traditional Chinese and Japanese medicine Ko-jo-kon (Nonomura et al., 1963). Resveratrol is present in skin and seeds of more than 70 different plant species, including grapes, berries, grains, tea, and peanuts (Soleas et al., 1997; Chen et al., 2002). In the presence of an enzyme resveratrol synthase, the phytochemical resveratrol is synthesized in response to environmental stress such as heavy metal ions, injury, fungal infection, or UV irradiation from coumaroyl CoA and malonyl CoA (Singh et al., 2013). It is synthesized in the pericarp of grape berries, epidermis of grape berry leaf, and in the stalks and kernels of the berries (Creasy and Coffee, 1988). It constitutes one of the primary components in red wine and is claimed to be an essential factor in the French Paradox, a term frequently used to summarize the apparently paradoxical epidemiological observation that French people have a relatively low incidence of CHD despite having a diet relatively rich in saturated fats (Liu et al., 2007; Sun et al., 2008). The level of resveratrol in plants reaches its peak approximately 24 h after stress exposure and subsides after 42–72 h due to the activation of stilbene oxidase (Soleas et al., 2001; Jeandet et al., 2002). Resveratrol belongs to a group of compounds called the stilbene family, which contain two aromatic rings joined by a methylene bridge. Stilbene synthase (STS), which belongs to a multigene family of the type 3 group of the polyketide synthase superfamily, is the enzyme that controls the production of resveratrol in plant tissues (Bais et al., 2000). Resveratrol exists in two geometric isomers with *trans* and *cis* configuration (**Figure 1**). *Trans*-resveratrol is considered to be a non-toxic potential stereoisomer and is widely known to possess the reported beneficial health effects (Orallo, 2006).

Indeed, resveratrol is also protective against oxidative stress, inflammation (Das and Das, 2007), and the development of cardiovascular diseases (Chen et al., 2002), diabetes (Venturini et al., 2010), neurodegenerative diseases (Vingtdeux et al., 2008), and cancer (Kris-Etherton et al., 2002). Resveratrol plays a prominent role in the prevention of neurodegenerative diseases such as AD, Parkinson’s disease, cerebral ischemia as well as Huntington’s disease because resveratrol enters the blood stream after the formation of glucuronide conjugates and can readily pass through the BBB; Baur and Sinclair, 2006). Resveratrol (10–100 μM) is reported to exert neuroprotective effects in several studies (Richard et al., 2011). In this review, we discuss the several beneficial effects of resveratrol and newly designed resveratrol analogs in AD and its potential to promote human health.

## METABOLISM AND BIOAVAILABILITY OF RESVERATROL

Resveratrol is rapidly absorbed and metabolized on oral administration to form glucuronide and sulfate conjugates, which are excreted in urine (Vingtdeux et al., 2008). In humans, the primary metabolite of resveratrol is *trans*-resveratrol-3-*O*-glucuronide, whereas in mice and rats, *trans*-resveratrol-3-*O* glucuronide and *trans*-resveratrol-3-sulfate are the primary metabolites, respectively (Yu et al., 2002). Several studies conducted on the bioavailability of resveratrol indicate that poor absorption and rapid metabolism of resveratrol and its metabolites like glucuronides and sulfates results in low oral bioavailability of resveratrol (Wenzel and Somoza, 2005). Studies by Walle (2011) have shown the oral absorption rate of resveratrol to be 70–75% with respect to the urinary excretion of the total metabolites after radiolabelled doses. Plasma concentrations of resveratrol ranged from 1 to 5 ng/ml on administration of 25 mg resveratrol (Almeida et al., 2009), a concentration commonly used in experimental studies and associated with various beneficial effects on cardiovascular, endothelial, and neurologic function (Zhang et al., 2009, 2010; Clark et al., 2012; Rege et al., 2013). Administration of higher doses up to 5 g led to a proportional increase in the plasma resveratrol to about 500 ng/ml (Boocock et al., 2007). Despite its poor bioavailability and rapid disposal, resveratrol does indeed accumulate in tissues, including brain, even after acute or short-term treatment. Acute administration of resveratrol by oral gavage using a low dose of 80 μg/kg results in significant accumulation in brain within 4 h (Bertelli et al., 1999). Short term treatment using a concentration of 40 μg/kg by the same route of administration for a period of 15 days also increases resveratrol content in the brain (Bertelli et al., 1999). Resveratrol is known to have several beneficial effects in brain but its poor bioavailability or the matrix within which it is contained in the dietary media are issues of major concern for resveratrol delivery (Goldberg et al., 2003; Mohar and Malik, 2012).

## BIOAVAILABILITY OF RESVERATROL AND ITS ANALOGS

The BBB is a highly selective permeable endothelial cell layer connected by tight junctions, sequestering the CNS tissue from vasculature. This barrier is permeable to the flow of water, some gases, nutrients, and lipid soluble molecules through passive diffusion. In addition, the BBB prevents entry of many neurotoxins by P-glycoprotein-mediated active transport. Polyphenols are accessible and enter the brain only if they cross the BBB (Vauzour, 2012). Based on *in vitro* studies, the permeability of polyphenols through the BBB depends on several factors such as the lipophilic state of the compound and increased capability of brain uptake by less polar polyphenols or metabolites (such as *O*-methylated derivatives; Youdim et al., 2003). Resveratrol being a lipophilic compound can readily cross the BBB via transmembrane diffusion (Lin et al., 2010). Further, to effectively penetrate the BBB, molecules must be below 500 Da in molecular weight (Banks, 2009). Resveratrol, with its molecular weight of 228 Da (Amri et al., 2012) and lipid soluble properties, should easily cross the BBB. Faria et al. (2010) demonstrated that similar sized flavonoids found in red wine such as quercetin and catechin can easily penetrate membranes in RBE-4 cells, an immortalized cell line of rat cerebral capillary endothelial cells.

However, resveratrol’s low bioavailability originating from its poor water solubility and resulting from its short biological half-life, labile properties, rapid metabolism and clearance limits the efficacious concentrations of resveratrol to accumulate in plasma and target tissues (Walle, 2011; Cho et al., 2014). Therefore, several drug delivery systems are designed to improve these inherent biologic limitations of resveratrol, such as increasing its solubility and preventing resveratrol from rapid degradation while preserving its biological activity. Approaches aimed at controlling its release from the gastrointestinal tract to enhance its bioavailability are also considered (Sessa et al., 2011; Augustin et al., 2013). At present, several drug delivery systems for enhancing the bioavailability and solubility of resveratrol have been developed such as encapsulation in liposomal formulations, the design of resveratrol–protein complexes to favor resveratrol binding to protein, use of cyclodextrin complexes and solid lipid nanoparticles for enhanced matrix-based delivery, pectinate delivery systems, and chitosan microspheres (Augustin et al., 2013). In agreement with this novel delivery systems, recent evidence has shown that administration of 5 mg/kg of resveratrol in loaded-lipid core nanocapsules every 12 h intraperitoneally for 14 days is effective against the neurotoxicity induced by intracerebroventricular injection of Aβ1–42 in rats (Frozza et al., 2013). Also, the use of more potent analogs of resveratrol such as SRT501 (Howells et al., 2011) and resveratrol in combination therapy with piperine, a natural product obtained from black pepper, have proved to be efficient methods of enhancing its bioavailability (Johnson et al., 2011).

Recently, Csiszár et al. (2014) reported that encapsulation of resveratrol into novel fusogenic liposomes is more efficient than conventional liposomes. This approach enhances the delivery of polyphenol resveratrol into aged cells leading to the activation of cellular Nrf2-mediated antioxidant defense systems (Csiszár et al., 2014). Furthermore, the use of polyethylene glycol (PEG) derivatives presents several advantageous features for delivery. PEG as an oral vehicle material is a non-toxic polymer, has high water solubility, is both non-teratogenic and non-immunogenic, and exhibits antigenic properties. Two types of amino acid PEGylated resveratrol conjugates developed to date demonstrate increased solubility of resveratrol up to 900 mg mL^-1^, clearly highlighting the potential of PEGylated compounds as an effective system for improving the solubility and bioavailability of resveratrol (Zhang et al., 2014).

Resveratrol exhibits good absorption rates but low bioavailability. An oral dose of 25 mg results in less than 5 μg/mL in the serum following absorption through the gastrointestinal tract, corresponding to approximately a 1000-fold decrease in bioavailability. A 125-fold lower dose of 0.2 mg, yet in the milligram amount, injected intravenously results in plasma levels in the low nanogram range (16.4–30.7 ng/mL) (Walle et al., 2004) This rapid clearance is due to the reduction in the amount of free resveratrol from conjugation by sulfation and glucuronidation via P 450 enzymes. Albumin and lipoproteins serve as the major carriers for resveratrol in plasma (Delmas et al., 2011). After ingestion of resveratrol, conjugation produces resveratrol-*O*-glucuronide and resveratrol-*C*-glucuronide (Cottart et al., 2010). More than 90% of free resveratrol is bound to human plasma lipoproteins and 50% of the plasma *trans*-resveratrol-3-sulfate, *trans*-resveratrol-disulfates and the novel *trans*-resveratrol-C/O-diglucuronides are non-covalently bound to proteins as reported by Burkon and Somoza (2008). Evidence shows that resveratrol undergoes enterohepatic metabolism in both rats and humans (Timmers et al., 2012). After uptake by enterocytes, resveratrol is metabolized to sulfate and glucuronide conjugates, which may be deconjugated by gut microflora, reabsorbed, or excreted in the feces. Thus, the enterohepatic circulation decreases the amount of free compound reaching the target tissues. Hence, a small fraction of the ingested resveratrol reaches the tissues (Timmers et al., 2012). In a brain ischemic model, results suggest that resveratrol enters the blood stream after its formation to glucuronide conjugates and could thereby cross the BBB (Wang et al., 2002). To overcome the potential limitations of low bioavailability and metabolism, a therapeutic approach in developing congeners and analogs of resveratrol should be undertaken. Modification of resveratrol’s chemical structure by altering the number and position of the hydroxyl groups, intramolecular hydrogen bonding, double bonds, and stereoisomerism is crucial for improving the efficacy and enhancing the bioavailability. Stilbene monomers include methyl and methoxy group subsitutions, and variations in *cis* and *trans* configurations (Cottart et al., 2010). Systemic exposure to pterostilbene, a dimethyl derivative of resveratrol, resulted in significantly higher plasma levels when compared with resveratrol following administration at equimolar doses in male rats over 14 consecutive days. Treatment with pterostilbene also produced a sevenfold rise in its oral bioavailability than the parent resveratrol (Kapetanovic et al., 2011). A recent study by Greer et al. (2014) aimed at improving the bioavailability of *trans* resveratrol (tRes) by modifying its structure to reduce glucuronidation revealed three new stilbene derivatives. These derivatives of tRes with hydroxyl, aromatic nitro and carboxyl substituents to create NI-ST-05, DNR-1, and NI-12a, respectively, were less prone to glucuronidation, suggesting that tRes analogs improve bioavailability and could be potentially developed as alternate therapeutics (Greer et al., 2014). Several other resveratrol analogs such as hexahydroxystilbene (M8), galic acid, digalloyl resveratrol exert free radical scavenging properties and anti-carcinogenic effects (Szekeres et al., 2010). Another naturally occurring resveratrol analog, 3,5,4′-trimethoxy-*trans* stilbene, had greater plasma exposure, a longer half-life and lower clearance rates in rats (Lin and Ho, 2009). Several studies have indicated resveratrol to be a potent activator of SIRT 1. Sirtuins are NAD^+^-dependent class III histone/protein deacetylase (HDAC) enzymes. SIRT 1 deacetylates nucleosomal histones at specific residues by translocating from cytoplasm to nucleus and contributes to transcriptional silencing of telomeres and life span expansion (Pallàs et al., 2013). Recent studies demonstrate that both natural and synthetic sirtuin activating compounds (STACs) promotes allosteric SIRT 1 activation by binding of STACs to a conserved N-terminal domain in SIRT1. Recently sirtuins have gained considerable importance due to its key role in the calorie restriction (CR) response and as possible therapeutic drug targets. Amongst all the naturally occurring activators of SIRT 1, resveratrol is considered to be the most effective SIRT 1 activator. However, synthetic STACs have been documented to possess more potency, solubility and bioavailability as compared to natural STACs. The first synthetic STACs such as SRT1460, SRT1720, and SRT2183 were derivatives of an imidazothiazole scaffold and chemically different from the polyphenol resveratrol. Like resveratrol, SRT1720 compound was shown to activate SIRT1 by lowering the *K*_m_ for the substrate peptide. The third generation STACs derived from benzimidazole and urea-based scaffolds were more potent than resveratrol itself (Hubbard and Sinclair, 2014)

## RESVERATROL ANALOGS IN THE TREATMENT OF AD

Currently, several studies have reported various polyphenols exhibiting neuroprotective effects both *in vivo* and *in vitro*. Resveratrol and its derivatives have gained a prime importance amongst all these polyphenols due to their neuroprotective properties.

Piceatannol, a monohydroxylated derivative of resveratrol that differs by an additional hydroxyl group in 3′ of benzene ring, has shown to exhibit neuroprotective effects against beta-amyloid induced neural cell death by blocking Aβ-induced accumulation of ROS (Kim et al., 2007b). Pterostilbene has shown to be a potent modulator of cognition and cellular oxidative stress associated with AD (Chang et al., 2012). In addition to monomers, several dimers and oligomers have been developed. Two new stilbene dimers, scirpusin A with an additional hydroxyl group, and ε-viniferin glucoside, with a glucose moiety, demonstrated a robust inhibition of fibril accumulation, thereby could be used as efficient fibril inhibitors in the treatment of AD (Rivière et al., 2010).

Lu and colleagues designed a novel series of resveratrol derivatives serving as multi-target agents in the treatment of AD. Amongst the synthesized compounds, 5d (E)-2-((4-(3,5-Dimethoxystyryl) phenylamino) methyl)-4-(dimethylamino) phenol and 10d (E)-5-(4-(5-(Dimethylamino)-2-hydroxybenzylamino) styryl)-benzene-1,3-diol exerted significant inhibition of Aβ aggregation, metal-chelating ability, disintegration of highly structured Aβ fibrils and Cu(II)-induced Aβ aggregation, antioxidant activity and low neurotoxicity. Moreover, compound 5d could also cross the BBB *in vitro* and doses up to 2000 mg/kg were not associated with any signs of toxicity in mice (Lu et al., 2013). Lu and colleagues previously reported a series of stilbene derivatives based on the structure of resveratrol in which compound 7l (E)-5-(4-(isopropylamino)styryl)benzene-1-3-diol exerted potent β-amyloid aggregation inhibition activity (Lu et al., 2012). Novel synthetic compounds such as STACs confer remarkable health benefits in various animal models. SRT3025 is one such STAC, which penetrates the BBB; mimics the effects of CR on the brain and further reduces neurodegeneration (Hubbard and Sinclair, 2014). In conclusion, various resveratrol analogs developed with improved bioavailability possess neuroprotective properties and could be further used as novel multifunctional drugs in the treatment of AD.

### RESVERATROL CONTENT IN WINE AND PLANTS

Resveratrol occurs as free resveratrol and as 3 β-glucoside, a derivative of resveratrol both in grapes and wine (Vrhovšek et al., 1995; Romero-Pérez et al., 1996). Concentrations of resveratrol in grape species range from 50 to 400 μg/g fresh weight in the leaves and fresh grape skin contains around 50–100 μg of resveratrol per gram (Jeandet et al., 1991). In grape juices, the concentration of free resveratrol is low as compared to *cis*- and *trans*-piceid derivatives of resveratrol (Romero-Pérez et al., 1999). The levels of resveratrol vary from 3 to 15 μg/L and 690 to 14,500 μg/L in grape juices (Romero-Pérez et al., 1999).

The concentration of resveratrol in wine varies considerably and is also largely dependent on the grape cultivar, geographic conditions and exposure to fungal infections. Typically, the total concentration of resveratrol in red wine is between 0.2 and 5.8 mg/L while white wine contains approximately 0.68 mg/L. Red wines have six times higher concentrations of *trans*-resveratrol than white wines while white wines contain high levels of *cis*-resveratrol. Red wine is extracted without removing the grape skin, whereas white wine is fermented only after removal of the skin (Prasad, 2012). Other sources of common foods containing resveratrol include dark chocolate, various berries, soy, and raw or boiled peanuts.

### ANTIOXIDANT PROPERTIES OF RESVERATROL IN AD

Resveratrol exhibits strong antioxidant properties as shown by *in vitro* and *in vivo* studies (Sönmez et al., 2007; Venturini et al., 2010). Oxidative stress occurs due to an imbalance between pro-oxidant and antioxidant activities in the body leading to the excessive production of ROS, free radicals and peroxides (Barnham et al., 2004). Brain tissue is more susceptible to oxidative stress due to its greater rate of oxygen consumption, high content of peroxidizable fatty acids, less regenerative capability, and low amounts of antioxidants. Thus, free radicals seem to play a crucial role in the process of brain aging (Floyd, 1999; Honda et al., 2004; Romano et al., 2010). AD is an age-related disorder, most often diagnosed in individuals over 65 years of age and hence aging is strongly implicated in the pathogenesis of this disease (Jayasena et al., 2013).

Alzheimer’s disease is characterized by neuritic plaques composed of insoluble deposits amyloid β peptide (Vingtdeux et al., 2008), neurofibrillary tangles and synaptic loss together, which leads to a gradual decline in cognitive function (Kolarova et al., 2012). The hallmarks of AD are the presence of neurofibrillary tangles and Aβ senile plaques in the cortex and the hippocampus, respectively (Selkoe, 2002). Hyper-phosphorylation and abnormal deposition of tau protein results in the formation of neurofibrillary tangles whereas Aβ senile plaques contains deposits of β-amyloid (Aβ) peptide (Golde et al., 1992). Beta-amyloid is a 39–43 amino acid peptide fragment derived from the sequential proteolytic cleavage of the APP by the enzymes beta (β) and gamma (γ) – secretase (Huang et al., 2011). In 2000, around 25 million people were diagnosed with AD worldwide, and this number is expected to increase to 114 million by 2050 (Wimo et al., 2003). Early age onset AD is a form of AD diagnosed in the age group younger than 65 years. A small portion of all early age onset AD population consists of familial AD cases whereas a large portion of late onset AD patients are sporadic AD cases, a form of AD diagnosed in the population older than 65 years (Piaceri et al., 2013). In familial AD patients, mutations are observed in the APP, presenilin 1 (PSEN1), and presenilin 2 (PSEN2) genes. Though the specific causes of sporadic AD are unknown, many genetic and environmental factors contribute to the development of sporadic AD (Selkoe, 2001). The key factors contributing to the pathogenesis of both familial and sporadic forms of AD are Aβ peptides (Selkoe, 2001; Selkoe et al, 2004). Thus, the therapeutic goal in the treatment of AD serves to target both Aβ production and amyloid fibril aggregation (Roberson and Mucke, 2006). Oxidative stress caused by an excessive production of ROS in the brain has been considered as the underlying cause for the pathogenesis of a number of neurodegenerative disorders. An increase in levels of ROS, reactive nitrogen species, or some malfunction of the cellular antioxidant systems can damage protein and membrane poly unsaturated fatty acids, causing lipid peroxidation and further leads to loss of membrane integrity and increased permeability to Ca^2+^ in the plasma membrane (Floyd, 1999; Sun et al., 2008; Rege et al., 2013). Moreover, it causes injury to neural membranes and ultimately memory impairment (Sun et al., 2010). Several *in vivo* and *in vitro* studies have reported that ROS increases Aβ production and Aβ induces oxidative stress, which may together accelerate the progression of AD (Murakami et al., 2005; Tabner et al., 2005). However, plant derived dietary antioxidants can be regarded as potential useful targets for the prevention of neuronal damage in neurodegenerative disorders.

**FIGURE 2:**
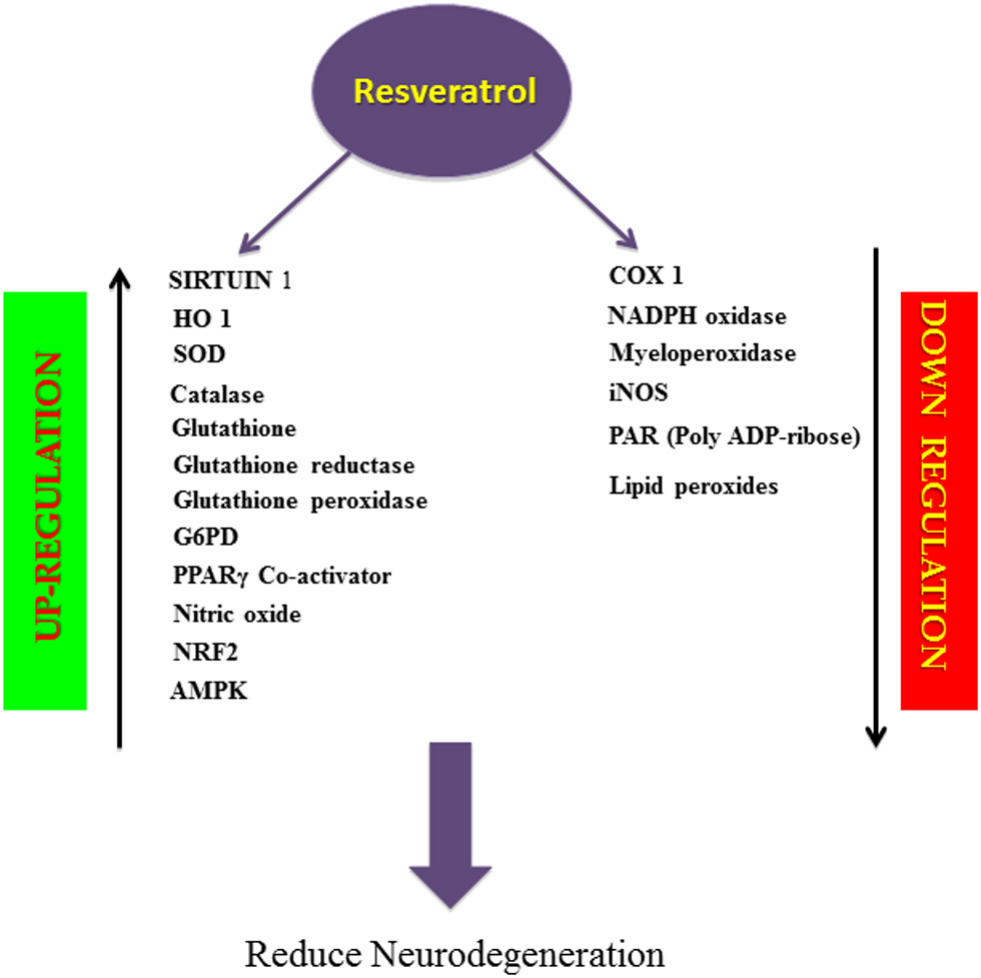
**Schematic overview of biological activity of resveratrol.** Resveratrol, a natural antioxidant, upregulates the activity of SIRT1, enzymatic antioxidants, PPARγ Co-activator, NO, NRF2, AMPK and downregulates the activity of COX1, NADPH oxidase, Myeloperoxidase, iNOS, PAR, Lipid peroxides thereby prevents apoptosis and inflammation and reduces neurodegeneration.

Resveratrol suppresses oxygen free radical formation by inhibiting pro-oxidative genes such as nicotinamide adenine dinucleotide phosphate oxidase and myeloperoxidase, and inducing various antioxidant enzymes like SOD, catalase, thioredoxin and glutathione peroxide (GSH-Px; Wang et al., 2012; Carrizzo et al., 2013), while lowering the activity of enzymes involved in the development of oxidative stress (Carrizzo et al., 2013; **Figure 2**). Thus resveratrol is a direct scavenger of free radicals production in tissues. It is interesting that resveratrol has proven to be effective in suppressing iNOS production, which is involved in the Aβ-induced lipid peroxidation and heme oxygenase-1 (HO-1) downregulation, thereby protecting the rats from Aβ-induced neurotoxicity (**Table 1A**; Huang et al., 2011; **Figure 2**). One of the major pathological features in AD is cerebral metal ion imbalance. Ions of copper, iron, zinc, and aluminum act as key cofactors in various neuronal functions, including cellular respiration, cellular redox homeostasis, nerve transmission, oxygen transport and functioning of the channels. Dysregulation in the metal ion balance plays a key role in driving neurodegeneration, which is likely to impact cellular function and ultimately neuronal survival. Decreased levels of copper lead to ROS generation and neuronal inflammation in association with Aβ deposition. Resveratrol administration with a high affinity copper chelator may attenuate copper imbalance and ROS production. Similarly, resveratrol can prevent the accumulation of free iron and iron mediated ROS generation and can also counteract the iron-induced mitochondrial dysfunction by suppressing GSK3β activity. Excessive accumulation of zinc and aluminum also promotes ROS production, increases neuroinflammation eventually leading to AD. However, resveratrol has not shown to have direct effects on the levels of zinc but can prevent further development of zinc-related ill effects. Moreover, resveratrol seems to cause an ameliorative change in aluminum induced neurotoxicity (Granzotto and Zatta, 2014). Findings of Granzatto et al. suggest that resveratrol acts as a neuroprotectant against Aβ as well as against Aβ-metal complexes. In addition, resveratrol exerts ROS scavenging properties and reduces toxicity against Aβ-Fe, Aβ-Cu, and Aβ-Zn, but fails to completely block Aβ-Al and Aβ-Cu toxicity (**Table 1B**; Granzotto and Zatta, 2011). A substantial amount of research has attributed this polyphenol for its anti-antioxidant and cytoprotective actions in oxidative stress-induced brain pathologies. Consequently, resveratrol appears to improve glial, oxidative and inflammatory responses by enhancing the expression of HO-1 and extracellular GSH content in H_2_O_2_-induced C6 cells (Quincozes-Santos et al., 2013). Moreover, resveratrol also protected PC12 cells against amyloid-induced cytotoxicity, cell death, and intracellular ROS accumulation and also suppressed beta-amyloid-induced activation of NF-KB in PC12 cells (Jang and Surh, 2003). Another key enzyme known as Poly (ADP-ribose) polymerase-1 (PARP-1) plays a key role in the regulation of Aβ precursor protein metabolism processing. Studies have reported that over-activation of PARP-1 due to oxidative stress leads to an accumulation of the novel signaling molecule poly-ADP-ribose (PAR), which induces neuronal cell death associated with AD pathogenesis (Strosznajder et al., 2012; **Figure 2**). Findings by Lee et al. indicated resveratrol reduced PARP-1 cleavage and protected SH-SY5Y neuroblastoma cells from apoptosis (Lee et al., 2007). Resveratrol being a robust activator of SIRT1 has shown to possess anti-amyloidogenic activity through the activation of SIRT 1 in the brains of Tg2576 mice and protects the cells against oxidative damage (Kelsey et al., 2010; **Figure 2**) Furthermore, resveratrol prolongs the synthesis of Aβ in neuronal cultures expressing APP and reduces Aβ production by stimulating SIRT 1 activity (Tang and Chua, 2008). Also, resveratrol protects neocortical neurons cultured from the senescence-accelerated mouse strain SAMP8 against increased susceptibility to oxidative damage via SIRT 1 activation (**Table 1C**; Cristòfol et al., 2012). Thus, SIRT 1 appears to be a promising new avenue for therapeutic intervention in age related AD.

**Table 1:** Neuroprotective effects of resveratrol in animal models and cultured cell systems.

Experimental animals	Model	Effects	Dosage	Treatment/time of incubation	Reference
(A) Sprague–Dawley Rats	AD	Decreased hippocampal Aβ accumulation Improved Aβ-induced spatial memory Reversed Aβ induced iNOS expression Enhanced HO 1 expression and reduced lipid peroxidation	100 μM/5 μl	7 days for 30 min	Huang et al. (2011)
(B) SH-SY5Y human neuroblastoma	–	Protected against Aβ as well as Aβ metal complexes. Acts as a ROS scavenger. Reduced Aβ and Aβ metal complex toxicity.	15–200 μM for 24 h	Aβ and Aβ complexes	Granzotto and Zatta (2011)
(C) Neocortical neurons – SAMP8 mice	–	Increased expression of SIRT 1 Decreased susceptibility to oxidative damage.	50 μM for 2 h and 24 h	BSO (DL-Buthionine-sulfoximine)	Cristòfol et al. (2012)
(D) APP/PSI transgenic mice	AD	Decreased Aβ-associated microglial activation. Lowered cerebral amyloid deposition	350 mg/kg BW	15 days	Capiralla et al. (2012)
(E) Senescence accelerated mice models	AD	Increased antioxidant status and decreased lipid peroxidation. Prevents cerebral mitochondrial deletion. Increased learning and memory impairment.	25, 50, 100 kg^-1^d^-1^	8 weeks	Liu et al. (2012)
(F) p. 25 transgenic mouse	AD	Decreased hippocampal neurodegeneration Increased cognitive performance Decreased acetylation of SIRT 1 substrates PGC-1 alpha and p53	5 μg/μl injected bilaterally	2–3 times/week	Kim et al. (2007a)
(G) PC 12	–	Inhibited β-amyloid-induced cell apoptosis. Up regulated SIRT 1 levels. Down-regulated ROCK 1 expression.	12.5–100 μM for 24–48 h	Amyloid-β	Feng et al. (2013)
(H) APP-HEK_293_ and APP-N_2a_	–	Lowered Aβ levels by activating AMPK pathway. Induced autophagy and lysosomal degradation of Aβ	40 μM for 24 h	Aβ_1-40_ and Aβ_1-42_	Vingtdeux et al. (2010)
(I) Tg19959 transgenic mice	AD	Reduced plaque counts and burden in medial cortex, striatum and hypothalamus Increased brain cysteine levels to 54% and decrease in brain glutathione levels to 21%.	300 mg/Kg BW	45 days	Karuppagounder et al. (2009)
(J) SAMP8 mice	AD	Increased life expectancy. Decreased cognitive impairment. Increased both SIRT 1 and AMPK levels. Decreased P53 acetylation. Reduced amyloid deposition and favored non-amyloidogenic pathway in hippocampus.	1 g/kg BW	7 months	Porquet et al. (2013)
(K) Wistar rats (Colchicine-induced)	AD	Decreased cognitive impairment Decreased lipid peroxidation and nitrite levels Increased acetylcholinesterase activity Restoration of GSH levels	10–20mg/kg	25 days beginning 4 days prior to colchicine injection	Kumar et al. (2007)
(L) Primary cortical neurons from mice	–	HO1 production acts as neuroprotection	5–100 μM for 6 h	Resveratrol alone	Zhuang et al. (2003)

### BENEFICIAL EFFECTS OF RESVERATROL ON NEURONAL INFLAMMATION IN AD

Neuronal inflammation promotes the pathogenesis of several chronic neurodegenerative diseases, including AD. Various reports show that the inflammatory responses occurring in central nervous system such as activation of microglia, astrocytes, lymphocytes and macrophages triggers numerous pro- and anti-inflammatory mediators such as ROS, NOS, cytokines, and various neurotransmitters (Moore and O’Banion, 2002). Activation of microglia releases highly ROS such as hydroxyl radicals, superoxide and per oxy radicals, hydroxyl peroxide, and thereby causes oxidation of proteins, lipid peroxidation, and DNA fragmentation. These processes eventually lead to neuronal inflammation and cell death (Liu and Hong, 2003). Amyloid β peptides, the major component of amyloid plaques interact with various Toll-like receptors (TLRs) such as TLR4 and can trigger microglial activation. Anti-inflammatory action of resveratrol has shown to prevent lipopolysaccharide (LPS, a TLR4 ligand)-induced activation of murine RAW 264.7 macrophages and microglial BV-2 cells. It also prevented proinflammatory effect of Aβ on macrophages by inhibiting activation of STAT 1 and STAT3 and NFκB activation by interfering with IKK and IκB phosphorylation (Capiralla et al., 2012). In addition, oral administration of resveratrol in a mouse model of cerebral amyloid deposition significantly reduced microglial activation related to amyloid deposition (**Table 1D**; Capiralla et al., 2012). Since NF-κB signaling is involved in Aβ-induced neuronal cell death, another link between AD and neuroprotective action of resveratrol is its potential to decrease the expression of iNOS, prostaglandin E2 (PGE2), cathepsin and NO modulated by NF-κβ (Kim et al., 2006). Lu and colleagues reported that resveratrol attenuates LPS-stimulated NF-κB activation in murine primary microglia and astrocytes and LPS-induced inflammatory responses could be modulated by different potencies of resveratrol (Lu et al., 2010). Studies have shown that astrocytes in brain have both positive and negative effects on the central nervous system. They serve as a source of nutrients to neurons and aid in the maintenance of extracellular ion balance as well as in the clearance and degradation of Aβ (Wyss-Coray et al., 2003; Lee et al., 2010). Astrocytes also secrete prostaglandins, interleukins, leukotrienes, thromboxanes, and form bunches around Aβ deposits (Sidoryk-Wegrzynowicz et al., 2011). A study by Simao et al. showed resveratrol pretreatment (30 mg/kg) significantly reduced NF-κB and JNK activation, and decreased the global cerebral ischemia-induced astroglial and microglial activation and iNOS and COX-2 regulation (Simão et al., 2012). Resveratrol reduces the concentration of 8-iso-prostaglandin F2α, an indicator of free radical production in LPS-activated rat microglial cells, and is considered to be involved in the downregulation of neuroinflammatory responses (Candelario-Jalil et al., 2007). Resveratrol treatment decreased lipid peroxidation, thereby causing an upregulation in the antioxidant status in the senescence-accelerated mouse model. It also prevented cerebral mitochondrial deletion and decreased the impairment in learning and memory (**Table 1E**; Liu et al., 2012).

### ANTI-AMYLOIDOGENIC EFFECTS OF RESVERATROL

Resveratrol exhibits its neuroprotective effects in the inhibition of β-amyloid production and aggregation and in the destabilization of the Aβ fibrils (Ono et al., 2006). Resveratrol also decreases the accumulation of Aβ in cell cultures and lowers Aβ secretion from different cell lines. Since it has no effect on the Aβ producing enzymes, β and γ secretases, it does not suppress Aβ production but promotes proteolytic clearance of Aβ through a mechanism that implicates a proteasome and not NEP (neprilysin) ECE-1 and ECE-2 (endothelin converting enzyme 1 and 2) or IDE (insulin degrading enzyme) (Marambaud et al., 2005). Chronic administration of resveratrol proved to be effective in protecting animal models of AD from Aβ-induced neuronal loss, cell death, accumulations of lipid peroxide products, inhibition of hippocampal iNOS production, and the elevation of HO-1 expression. In accordance with this, resveratrol showed recovery from Aβ-induced spatial memory impairment in the animal models of AD (Huang et al., 2011). Further, consumption of red wine significantly reduces the impairment of spatial memory function and Aβ neuropathology in Tg2576 mice (Wang et al., 2006). Another study by Lu et al. suggested that administration of resveratrol lowered MPTP-induced deterioration of motor coordination and neuronal loss caused by excessive production of free radicals (Lu et al., 2008). A marked reduction in neurodegeneration in the hippocampus was observed on administration of intracerebroventricular injection of resveratrol, which was caused by a decrease in the acetylation of SIRT1 substances such as peroxisome proliferator-activated receptor gamma co-activator and p53 (Kim et al., 2007a). This eventually prevented learning deficit in the p25 transgenic mouse model of AD (**Table 1F**; Kim et al., 2007a). Moreover, an *in vitro* model of PC12 cells using Aβ_25-35_ provided new compelling evidence on the protective effect of resveratrol against Aβ induced neurotoxicity. Resveratrol protected PC12 cells and inhibited Aβ-induced cell apoptosis through the upregulation of SIRT 1 expression and downregulation of Rho-associated kinase 1 (ROCK 1). Thus, anti-apoptopic actions of resveratrol were partially mediated through the SIRT1-ROCK 1 pathway (**Table 1G**; Feng et al., 2013). Resveratrol is also found to exert its neuroprotective actions via the activation of key metabolic sensor proteins, such as the AMP-activated protein kinase (AMPK; **Figure 2**). Resveratrol induced AMPK activation results in the inhibition of AMPK target mTOR (mammalian target of rapamycin), initiation of autophagy and promotion of lysosomal clearance of Aβ (Vingtdeux et al., 2010). Studies indicate that resveratrol lowers Aβ accumulation in the cortex due to activation of AMPK signaling by enhancing cytosolic Ca^2+^ levels and CaMKKβ-dependent phosphorylation of AMPK in primary neuronal cultures (**Table 1H**; Vingtdeux et al., 2010). It has also been shown to decrease the formation of plaques in specific regions of brain thereby slowing down the process of neurodegeneration (**Table 1I**; Karuppagounder et al., 2009). A recent study by Porquet and colleagues reported that dietary resveratrol supplementation at the dose of 1 g/kg body weight to SAMP8 mice, an age-related model of AD, activates AMPK pathways, prosurvival routes such as SIRT1 and reduces amyloid accumulation, tau hyperphosphorylation and cognitive impairment (**Table 1J**; Porquet et al., 2013). Resveratrol at dosages of 10 and 20 mg/kg manifests a neuroprotective action against colchicine-induced cognitive impairment and oxidative damage in Wistar rats (**Table 1K**; Kumar et al., 2007). Furthermore, resveratrol treatment has also shown to suppress the levels of NOS and the expression of COX-2 in beta-amyloid treated C6 glioma cells (Kim et al., 2006). Another key player in the regulation of cellular antioxidant mechanism is nuclear factor erythroid 2-related factor 2 (Nrf2). Nrf2 serves as a chief regulator of cellular resistance to oxidants and genes encoding antioxidant proteins such as HO-1, NAD (P) H-quinone oxidoreductase, GST and glutathione synthetase (GSS; Scapagnini et al., 2011). Under normal unstressed conditions, Nrf2 is anchored by Keap 1 (Kelch-like ECH-associating protein 1) in the cytoplasm, which causes polyubiquitination and proteasome mediated degradation. It has also been shown to induce HO1 via Nrf2 and PI3K/AKT pathways and thereby reduce ROS induced oxidative damage in PC 12 cells (Chen et al., 2005). Resveratrol is known to promote HO-1 expression through the activation of Nrf2 in primary neuronal cultures (**Table 1L**; Zhuang et al., 2003; **Figure 2**). Thus, Nrf2 serves as a promising target for resveratrol in the prevention/treatment of certain neurodegenerative diseases.

## CONCLUSION

Resveratrol has been recognized as a potential therapeutic agent for treating wide array of health conditions/diseases such as inflammation, pain, tissue injury, diabetes, and cancer. However, emerging evidence focuses strongly on its potential beneficial effects against several neurodegenerative diseases. In this review, we discussed the antioxidant properties as well as neuroprotective effects of resveratrol in the pathogenesis of AD. For example, in AD, resveratrol promotes clearance of Aβ peptides, anti-amyloidogenic cleavage of APP, its ability to reduce oxidative stress and neuronal cell death. Consequently, it is plausible to recommend resveratrol as one of the promising tools in the development of drug therapy for AD. Moreover, it is non-toxic, cost effective, and widely available. However, the efficacy and utility of resveratrol also depends upon its solubility and bioavailability. Therefore, future research on the design and synthesis of novel analogs needs to be conducted to address these issues.

## Conflict of Interest Statement

The authors declare that the research was conducted in the absence of any commercial or financial relationships that could be construed as a potential conflict of interest.
